# Accelerated Brain Atrophy, Microstructural Decline and Connectopathy in Age-Related Macular Degeneration

**DOI:** 10.3390/biomedicines12010147

**Published:** 2024-01-10

**Authors:** Jacques A. Stout, Ali Mahzarnia, Rui Dai, Robert J. Anderson, Scott Cousins, Jie Zhuang, Eleonora M. Lad, Diane B. Whitaker, David J. Madden, Guy G. Potter, Heather E. Whitson, Alexandra Badea

**Affiliations:** 1 Brain Imaging and Analysis Center, Duke University Medical Center, Durham, NC 27710, USA; jacques.stout@duke.edu (J.A.S.); jie.zhuang@duke.edu (J.Z.); david.madden@duke.edu (D.J.M.); 2 Radiology Department, Duke University Medical Center, Durham, NC 27710, USA; ali.mahzarnia@duke.edu (A.M.); rui.dai@duke.edu (R.D.); robert.j.andersn@duke.edu (R.J.A.); 3 Ophthalmology Department, Duke University Medical Center, Durham, NC 27710, USA; scott.cousins@duke.edu (S.C.); nora.lad@duke.edu (E.M.L.); diane.whitaker@duke.edu (D.B.W.); heather.whitson@duke.edu (H.E.W.); 4 Department of Psychiatry and Behavioral Sciences, Duke University Medical Center, Durham, NC 27710, USA; guy.potter@duke.edu; 5 Department of Medicine, Duke University Medical School, Durham, NC 27710, USA; 6 Department of Neurology, Duke University Medical Center, Durham, NC 27710, USA

**Keywords:** age-related macular degeneration, aging, diffusion MRI, connectomics, tractography, brain networks

## Abstract

Age-related macular degeneration (AMD) has recently been linked to cognitive impairment. We hypothesized that AMD modifies the brain aging trajectory, and we conducted a longitudinal diffusion MRI study on 40 participants (20 with AMD and 20 controls) to reveal the location, extent, and dynamics of AMD-related brain changes. Voxel-based analyses at the first visit identified reduced volume in AMD participants in the cuneate gyrus, associated with vision, and the temporal and bilateral cingulate gyrus, linked to higher cognition and memory. The second visit occurred 2 years after the first and revealed that AMD participants had reduced cingulate and superior frontal gyrus volumes, as well as lower fractional anisotropy (FA) for the bilateral occipital lobe, including the visual and the superior frontal cortex. We detected faster rates of volume and FA reduction in AMD participants in the left temporal cortex. We identified inter-lingual and lingual–cerebellar connections as important differentiators in AMD participants. Bundle analyses revealed that the lingual gyrus had a lower streamline length in the AMD participants at the first visit, indicating a connection between retinal and brain health. FA differences in select inter-lingual and lingual cerebellar bundles at the second visit showed downstream effects of vision loss. Our analyses revealed widespread changes in AMD participants, beyond brain networks directly involved in vision processing.

## 1. Introduction

Age-related macular degeneration (AMD) is one of the most common causes of legal blindness in older adults [1,2,3], and it estimated to affect up to 300 million individuals by 2040 [4]. AMD is associated with greater age-related cognitive decline compared to control populations without AMD [5]. Yet, the mechanisms explaining the greater cognitive decline in AMD have not been well documented. Investigating the dynamics of structural changes during aging may provide insights into the underlying mechanisms that lead to increased vulnerability to aging in AMD populations.

AMD is caused by the appearance and proliferation of large deposits made up of a core of glycoproteins [6], surrounded by other proteins (APOE, chaperone, inflammation proteins) and lipids, known as drusen, all congregating near the Bruch’s membrane [7]. Though a small amount of drusen is normal, excess drusen leads to the thickening of the Bruch’s membrane, causing the atrophy of retinal cells and loss of central vision [8]. Despite the availability of stabilizing treatments, damage to vision is progressive and irreversible, often resulting in legal blindness.

Besides its direct effects on vision, AMD has also been associated with greater cognitive decline relative to control populations, i.e., with lower results on standardized tests measuring verbal functioning, processing speed, working memory, visuospatial processing, and attention [5,9,10,11,12]. Interestingly, this decline is particularly strong for verbal fluency, independent from visual ability [9,13,14]. Using cross-sectional data from the same cohort as in this study, it was shown that AMD individuals, compared to peers with normal vision, exhibit brain connectivity differences for language and memory areas [15]. Reduced functional connectivity has been observed for the lateral occipital and visual cortex [16]. Regional volume reductions have been reported in the visual cortex and optic radiation, as well as for the frontal cortex [17]. It is not known whether AMD pathology causes changes to the brain (directly or indirectly), or whether these changes co-occur with AMD due to shared risk factors for other neurodegenerative conditions. This question can be addressed by investigating the spatial patterns of brain changes associated with AMD and its progression. This ability to measure change is a feature of the longitudinal design, as adopted in this study, relative to a cross-sectional design. Identifying commonalities with other neurodegenerative diseases may point to shared mechanisms and new therapies for populations with higher risk for cognitive decline and for dementia prevention [18,19,20].

Aging is a major risk factor for AMD, as well as for late-onset neurodegenerative diseases, such as Parkinson’s and Alzheimer’s disease [21]. Several studies have demonstrated associations between risk of AMD and Alzheimer’s disease (AD) [22,23,24]. In AMD, degeneration of the retina is associated with the accumulation of drusen, primarily composed of extracellular beta-amyloid and lipids [25,26,27,28]. AD patients have a higher incidence of drusen deposition in the retina compared to controls [29]. Both AMD and AD demonstrate histopathological accumulation of beta-amyloid, associated with microvascular changes and local inflammation prior to the appearance of clinical pathology [30,31].

Multiple studies have demonstrated that vision pathology may be an early indicator of abnormal brain aging and AD, and they have identified common risk factors, including the presence of toxic amyloid oligomers or deposits, as well as associated immune modulators [32,33,34,35,36]. Besides pathology, volume reduction was observed for the optic tract [15] and visual cortex. Moreover, changes in specific brain networks have been associated with differences in cognitive performance in AMD [15,37].

Age-related sensory deprivation may affect brain health [38], and there is epidemiological evidence suggesting an association between hearing loss and increased risk for dementia [39]. While some MRI studies of subjects with hearing loss have only identified a smaller primary auditory cortex [40], others have shown widespread structural and connectivity changes, supportive of brain reorganization [41], including in prefrontal brain regions [42]. Lack of visual stimulation alters both the gray matter of the visual cortex [43,44] and visual pathways [17,45,46,47]. In addition, cortical thinning and changes in relaxation parameters in the occipital cortex have been reported for central vision loss, with distinct patterns of more widespread cortical thinning in AMD versus localized gray matter changes in juvenile macular degeneration [48]. Most tract-based studies have focused on selected pathways. Here, we pursued an unbiased approach to reveal brain-wide connectopathies and sought to reveal novel biomarkers based on bundle analyses.

We hypothesized that the spatiotemporal patterns of brain reorganization in AMD during aging can reveal effects of additional neurodegenerative components and point to common mechanisms, underlining the potential of using the eye to predict brain biomarkers. A better understanding of the interactions between sensory deprivation and brain aging may lead to novel strategies to support successful aging. To begin to answer such questions, we have used diffusion-weighted imaging to characterize the dynamics of volume and microstructural and connectivity changes in AMD subjects relative to controls, over the duration of two years.

Given the scarcity of longitudinal AMD studies, our primary objective was to examine brain changes over time to determine whether AMD individuals exhibited accelerated brain volume loss or microstructural changes relative to controls or whether differences observed at baseline merely persisted. Our second objective was to identify which brain connections change over time in AMD, to provide a better understanding of the mechanisms behind the visual and cognitive changes associated with AMD, due to remote connectivity. We conducted novel connectome analyses to determine which regions demonstrated the greatest AMD-related structural differences and their evolution over time. Using novel tractography analyses, we explored the evolution of the resulting connections of interest and whether connectivity differences between AMD and control participants increased with time. Identifying regions and connections involved in cognitive decline can help better understand the mechanisms of AMD-related cognitive deficits.

## 2. Materials and Methods

### 2.1. Participants

Our study was approved by the Duke University Medical Center Institutional Review Board and includes 20 individuals with AMD (67% female, 61–89 years of age, M = 73.7 years, SD = 9.6 years) and 20 healthy control participants (61% female, 56–84 years of age, M = 72.9 years, SD = 7.5 years). Individuals with AMD were referred from the Duke University Eye Center; age-matched controls were recruited from the friends and family of participants with AMD and from recruitment registries maintained in the Duke Aging Center. The inclusion criteria for AMD individuals required participants to be over 50 years of age and to have a prior clinical diagnosis of either dry or wet AMD causing visual impairment (20/40 or worse) for at least 1 year. All participants (AMD and controls) were examined by an ophthalmologist or optometrist for AMD presence or absence, and individuals with secondary ocular conditions (e.g., cataracts, glaucoma) causing uncorrectable vision impairment were excluded. Controls had lens-corrected vision better than 20/40 in both eyes. In an interim chart abstraction conducted on AMD patients recruited for the larger study from which this MRI study was drawn, fewer than 5% had only dry AMD, while most had wet AMD and an AREDS stage of 4 in both eyes (39.5%) or just in one (55.8%); 37.5% of these in the right and 62.5% in the left eye. The chart abstraction used data from the nearest ophthalmology clinical encounter to the time of study enrollment. Among the 20 patients included in this MRI study, 12 (i.e., 60%) had similarly poor vision in both eyes, whereas 3 had worse vision in the right eye, and 5 had worse vision in the left eye. Participants had to be able and willing to undergo MRI (no MRI-incompatible prosthesis, pacemaker, no current pregnancy, etc.), right-handed, and willing to return 2 years later. All participants included in the study signed informed consents before the start of the study and participated in a first visit followed by a second one, approximatively 2 years later.

### 2.2. Imaging

Anatomical and diffusion-weighted images were acquired using a 3 Tesla GE Premier MR system (GE HealthCare, Chicago, IL, USA), equipped with a gradient capable of 30 mT/m strength and 150 T/m/s slew rate and an eight-channel head coil. Anatomical images were acquired to enable spatial normalization of subject images into an anatomical atlas, and produce a brain parcellation. Diffusion-weighted images were acquired to characterize microstructural tissue properties, including the degree of anisotropy, and quantify white matter fiber orientations, followed by tractography modeling of the streamlines connecting different brain regions and bundle analyses of streamlines clustered based on spatial proximity.

To produce a brain parcellation for connectivity analyses, we acquired anatomical images using a 3D Fast Spoiled Gradient Echo Sequence (FSPGR) [49] with the following specifications: TR = 8.16 ms; TE = 3.18 ms; TI = 450 ms; FOV = 25.6 cm^2^, flip angle = 12°; voxel size = 1 × 1 × 1 mm; 166 contiguous slices and SENSE factor = 2. To estimate texture information reflective of microstructural integrity and to construct tracts and connectomes, we acquired diffusion images using a 2D Spin-Echo/Echo-Planar imaging sequence, with the following specifications: TR = 9000 ms, TE = 85.6 ms; FOV = 25.6 cm^2^; flip angle = 90°; voxel size = 1 × 1 × 2 mm; 68 slices parallel to the AC–PC plane. We acquired 30 diffusion-weighted directions, with b = 1000 s/mm^2^, and 4 non-diffusion-weighted images, using a gradient table prescribed by the scanner. The same experimental protocol was used as in [15].

### 2.3. Image Analysis

The diffusion images were preprocessed using a pipeline [50], modified to incorporate Principal Component Analysis denoising [51,52] and to use BET [53] for brain masking. The co-registration and eddy current correction relied on ANTs [54]. The MRtrix toolbox [55] was used for creating diffusion parametric maps, i.e., fractional anisotropy (FA). Fractional anisotropy was chosen as it estimates the degree of microstructural tissue anisotropy, and thus provides information on the axonal diameter, fiber density, and degree of myelination.

For voxel-based analyses [56], the anatomical images were registered to the IIT human brain atlas [57] using SAMBA [58] and applying a series of rigid, affine, and diffeomorphic transformations derived using advanced normalization tools [54]. A minimum deformation template was generated to reduce individual participant biases. The minimum deformation template is constructed through an iterative process that brings each subject into a common space, following a sequence of affine and deformable registrations that requires minimum shape and intensity changes. All participants images were mapped into the space of the minimum deformation template based on diffusion-weighted images (DWI). The same registration was applied to the Jacobian images created from the deformation fields, and FA images, and the reverse registration was used to bring the atlas labels into the original subject space for morphometry (volume) and texture analyses (FA). Local volume changes were estimated based on the Jacobian of deformation field warps. Voxel-based analyses used the SPM toolbox [59], after smoothing images with a three voxels kernel, and corrected using cluster-based False Discovery Rate (FDR) at 0.05 level [60].

The MRtrix toolbox was used for tractography [55] with seeding done in gray matter voxels [61]. Streamline generation was carried out via the probabilistic method Second-order Integration over Fiber Orientation Distribution (FOD) [62] with an FOD cutoff of 0.05, a step size of 0.1 mm, a minimum length of 0.1 mm, a maximum length of 410 mm, and a 45° angle. Two million streamlines were generated per participant. The streamlines were then registered into the minimum deformation template space using the same rigid, affine, and warp transforms as applied to the corresponding participant images.

Brain connectomes were defined by the adjacency matrices describing the number of streamlines connecting all possible pairs of brain regions. These were built from the tractography streamlines and the IIT atlas, which defines 84 different gray matter regions [63].

To identify connections that explain variance associated with each of the discriminating traits in our study (age and diagnosis) we used Tensor Network PCA (TN-PCA) [64] as in [65], as this method has been shown to perform very well [66,67]. TNPCA was chosen as it takes into account the graph topological structure and can include multiple features, which increases its ability to explain the relationships between connectomes and traits. TN-PCA maps the high-dimensional tensor network data to low-dimensional space in which each component has an assigned weight. The weight assigned to each pair of regions reflects the maximum change networks in the reduced embedding space associated with increasing the trait score. Together with grouping the initial and 2-year follow-up visit scans, this allowed us to determine differences between age-matched individuals with and without AMD, and longitudinal changes (delta) in connectivity between these groups. Our results indicate which connections were most relevant for the different comparisons. The connections of interest (COI) determined by TN-PCA to be the most discriminating between the controls and AMD participants were retained for bundle analyses, which complement the connectome analyses and may provide novel and sensitive biomarkers.

Streamlines were spatially matched by registering the fibers with a rigid and affine transform, then sampled uniformly in 50 points and clustered into bundles using QuickBundles [68,69] with a filter size of 15 mm. The Fiber Coherence to Bundle (FBC) [70] was determined using DIPY, and bundle shape similarity was estimated via the distance between bundles. This was determined by the minimum average Euclidean distance between two bundles centroids [69]. We used the BUndle ANalytic values (BUAN) [71] to compares bundles as this metric encompasses bundle adjacency and shape similarity. BUAN estimates a composite similarity score based on the shape and distance between bundles. The FA values for the top connections were mapped to bundle centroids and analyzed using two-tailed *t*-tests, and *p*-value < 0.05 was considered significant. To compare streamlines between groups, we used mixed-effect linear models in R [72]. Such models heavily reduce the implicit bias associated with streamlines from the same subject. When comparing FA along bundles, we also included in the model the spatial identifier of a given point along the streamline as a fixed effect.

## 3. Results

### 3.1. Volumetric Changes in AMD Participants

**Figure 1:**
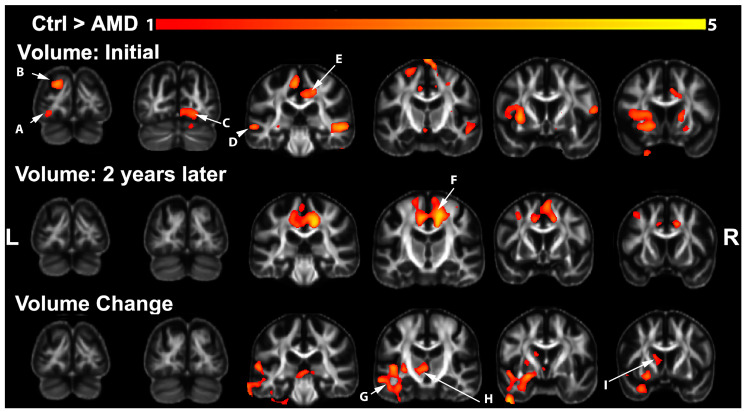
Brain regions showing reduced volume at the initial and second visit and an accelerated volume decline in AMD subjects. First row: differences at initial visit. Second row: differences at second visit. Third row: differences between rates of change. FDR-corrected (FDR = 5%) statistical parametric maps (t contrasts) are shown in color, overlaid on the minimum deformation template, shown using a grayscale colormap. Arrows: (A) left fusiform; (B) superior parietal/cuneus (top); (C) right lingual gyrus; (D) temporal gyrus; (E) posterior cingulate; (F) superior frontal; (G) left temporal gyrus; (H) thalamus; (I) caudate nucleus.

We identified widespread reductions in regional brain volumes in AMD participants relative to controls via voxel-based analysis (VBA) at the initial visit, two years later, and an accelerated rate of change between the two visits (Figure 1). At the initial scan time, we found lower volumes in the fusiform (A), the cuneus (B), and lingual gyri (C), which are involved in visual processing [73], as well as in the superior and middle temporal gyri (D), involved in language and memory processing. The left caudate-putamen, precentral and paracentral gyri, and bilateral posterior cingulate (E), involved in visual attention [74], also showed a pronounced reduction in AMD subjects at the first visit. At the second visit, differences between groups were identified in the bilateral posterior cingulate, paracentral, and superior frontal gyri (F). The rate of atrophy between the two time points was higher in AMD subjects, particularly for the left temporal lobe (G). The left thalamus (H) and dorsal striatum (I) also showed higher rates of atrophy in AMD.

### 3.2. FA Changes in AMD Participants

**Figure 2:**
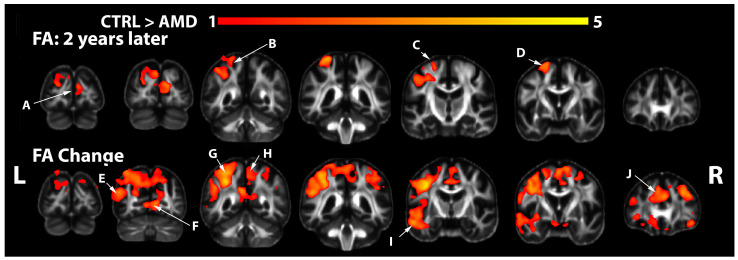
Fractional anisotropy reduction in AMD subjects relative to controls (FDR = 0.05). Significant FA reductions were observed at the second visit in areas including the following: (A) occipital lobe including the cuneus; (B) superior parietal and inferior parietal cortex; (C) precentral and postcentral gyri; (D) superior frontal. Accelerated loss of microstructural integrity, estimated via FA, was observed in the g: (E) inferior parietal; (F) pericalcarine, lingual cortex; (G) superior parietal, precuneus; (H) paracentral gyrus; (I) middle temporal/superior temporal; (J) superior frontal/anterior cingulate.

Compared to the morphometric results, we observed a more selective spatial pattern of FA reductions in AMD participants. At the initial visit, we found early FA reductions in the inferior temporal lobe of participants with AMD relative to controls in uncorrected statistics only, but these differences did not survive FDR correction. At the second visit, we observed extensive FA reductions for the cuneus (A), left superior and inferior parietal cortex (B), pre and postcentral gyrus (C), and superior frontal gyrus (D) (Figure 2). The FA rates of change revealed a role for the inferior and superior parietal cortices and precuneus (E, G); pericalcarine and lingual cortices (F), as well as for the paracentral (H); temporal (I); superior frontal, and cingulate cortices (J). We noted a lateralization that supports changes in the left temporal cortex (I), including Wernicke’s area associated with language. In conclusion, both memory and sensory-related brain areas, e.g., involved in vision, declined faster in AMD subjects relative to controls over a two-year period.

### 3.3. Connectivity Changes in AMD Participants

When comparing the connectomes of AMD and CTRL participants at the first visit, the only significant difference after FDR correction was between the insula and the rostral middle frontal gyrus (Table 1).

While other connections had nominally significant differences, given the high dimensionality of the connectomes, they did not survive an FDR correction. These included interhemispheric connections of the inferior temporal cortices, as well as the rostral middle frontal and post central gyrus. At the second visit, we noted the presence of the lingual cortex and cuneus involved in vision, as well as the latero-orbital frontal cortex, linked to cognitive functions, such as learning and reversing associations of visual and other stimuli with primary reinforcers; the superior parietal, linked to visuomotor and sensory and working memory processes; and pars opercularis, involved in language processing.

To reduce the high dimensionality of our connectome comparison problem, we performed TN-PCA at the first visit, 2 years later, and for the difference between these time points (Table 2). Our results at the first visit indicated that the connections that had the greatest weight in differentiating the AMD versus control participants were the interhemispheric lingual connection, followed by the fusiform right to superior temporal left, the superior frontal right to left, and the inferior temporal right to superior temporal left. At the second visit, the connections that influenced the TN-PCA analysis most heavily corresponded to the lingual gyrus to cerebellum connections, with a heavier weight for interhemispheric connections. We note that most of the subsequent connections involved either lingual or cerebellar regions.

Individuals with AMD demonstrated a greater loss of connections relative to controls within a two-year period, in particular those from the superior frontal left to the superior frontal right and to the rostral middle frontal right exhibited the largest group difference. Other connections with significantly greater loss in AMD subjects between the two visits involved the frontal regions or the lateral orbitofrontal and medial orbitofrontal regions. Three specific connections of interest were selected from those with high relevance during TN-PCA comparisons, including the following: Lingual Right–Lingual Left (LinR-LinL), Lingual Left–Cerebellum Cortex Right (LinL-CerebR), and Lingual Right–Cerebellum Cortex Left (LinR-CerebL).

Table 3 compares streamlines length and FA for three connections of interest (COI) determined by TN-PCA for the initial visit, and 2 years later. Differences in streamline length were significant for all COI, with CTRL participants having longer streamlines than those in the AMD group, while FA values did not show significant differences.

We compared spatially matched bundles resulting from streamline clustering. For the top ten largest bundles in all four groups (AMD and controls and first and second visit), we calculated the average distance of centroids and BUAN, where a higher BUAN score and lower average distance indicate that the bundles of a group are more similar in shape and show a lower spread. Table 4 shows the distance (spread), BUAN similarity, coherence, length, and FA of bundles for three COI. Group differences for distance in LinR-LinL were significant between AMD and control subjects at both visits, and this value decreased in AMD subjects between the two time points, but remained stable in controls. For BUAN, the LinL-CerebR differences were significant at the first visit and decreased in time for both LinL-CerebR and LinR-CerebL. The LinL-CerebR also showed major differences in coherence at the second visit, with AMDs showing lower coherence than controls.

Both Lingual–Cerebellar (Lin-Cereb) connections had a shorter length at the first visit in AMD, and the difference persisted for LinR-CerebL at the second visit and showed a similar trend for LinL-CerebR. Both the LinL-CerebR and LinR-CerebL bundles had significant differences in FA at both visits. While the LinR-LinL did not have significant differences at the first visit, it showed significantly higher FA values for the controls two years later. Thus, adding spatial specificity through bundle-matched analyses increased sensitivity to AMD effects on FA, at 5% FDR-corrected levels.

**Figure 3:**
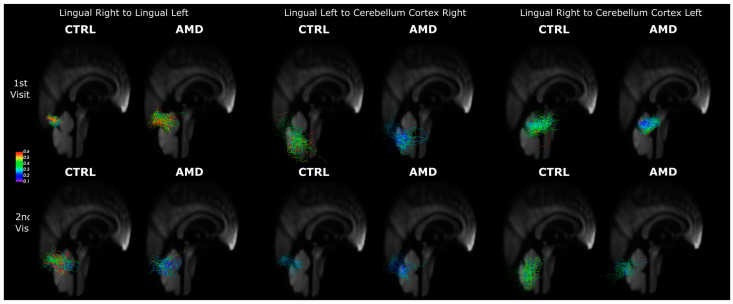
Bundles with greatest FA differences between AMD and controls for three connections of interest (COI), colored by FA, for the first visit (first row) and the second Visit (second row). First column: Lingual Right–Lingual Left; second column: Left Lingual–Cerebellum Right; third column: Lingual Right–Cerebellum Left).

Figure 3 shows bundle pairs with significant FA differences in Table 4 and that these were spatially equivalent in the common template space.

**Figure 4:**
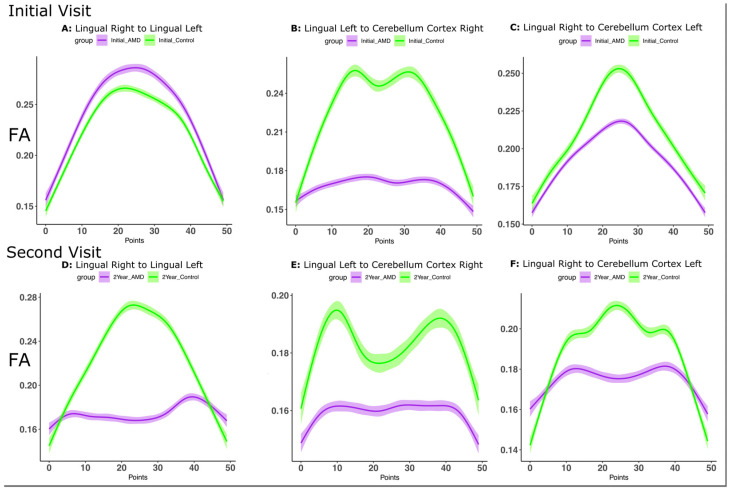
Bundle analyses for the connections determined by TN-PCA to contribute the most to the differences between AMD and CTRL participants at each visit. Initial visit: (**A**) Lingual Right–Lingual Left; (**B**) Lingual Left–Cerebellum Right; (**C**) Lingual Right–Cerebellum Left. Second visit: (**D**) Lingual Right–Lingual Left; (**E**) Lingual Left–Cerebellum Right; (**F**) Lingual Right–Cerebellum Left. Bundles were spatially normalized, and we established correspondence based on minimizing distances between bundle centroids.

Figure 4 shows FA profiles along the largest bundles in the control group compared to those that are closest to them in space and shape but found in AMD (as observed in Table 4). The interhemispheric lingual connections showed higher FA in controls for all bundles, except the LinR-LinL connection at the first visit (Figure 4B–F). While the FA was similar between groups at the first visit (Figure 4A), at the second visit, the control group displayed the same evolution of FA along the streamlines, while this was significantly reduced for AMD subjects (Figure 4D). The LinL-CerebR and LinR-CerebL bundle comparison showed significant group differences in both cases, and differences were similar between the two visits.

While the overall trend for the major bundles in the COIs was for decreased FA values in AMDs, we also noted local increases in FA in AMD subjects relative to CTRL, suggestive of possible compensatory remodeling of tracts.

## 4. Discussion

We tested if AMD, which is accompanied by sensory deprivation, contributes to or coexists with neurodegenerative processes in primarily visual and also remote brain areas. Our results have identified morphometric, microstructural, and connectivity changes based on longitudinal DWI in older adults with AMD relative to controls without AMD. Our study supports that connectopathies can provide important biomarkers for age-related neurodegenerative diseases and set the stage for developing models for the network-based propagation of diseases, to inform on future prognosis and the assessment of response to therapies.

Our results at the initial scan showed reduced volume in AMD subjects for the fusiform, lingual, cuneus, superior-parietal, and the temporal and cingulate gyri. The fusiform, cuneate, and lingual gyri are involved in processing visual information [73,75,76,77]. Changes in these areas of the occipital cortex were expected in patients with visual defects, as previous studies have demonstrated decreased occipital cortex volume both in early and late-onset blindness [78]. The cingulate cortex was shown to be involved in visual attention [74]. Both the (anterior) cingulate and temporal cortex have high connectivity and have been involved in cognitive processes, including language and memory [79,80].

Our results agree with fMRI studies showing that the temporal and lingual gyri have reduced functional connectivity in other forms of blindness [81,82], while the cuneate gyrus had atrophy [83]. Connectopathies in the fusiform, lingual, and temporal structures may result in visual and verbal memory dysfunction [84].

At the second visit, the cingulate and superior frontal cortex showed reduced volumes in AMD versus controls. These results are of interest as the central primary visual cortex has a particularly strong connection to the frontal cortex over the peripheral visual cortex [85] and AMD pathology particularly affects central vision.

In contrast to morphometry, the fractional anisotropy (Figure 2) showed no significant group differences at the first visit, but these were detected at the second visit in visual areas, the superior frontal gyrus involved with working memory, as well as the precentral and postcentral gyri involved in motor control and proprioception, the superior and inferior parietal cortex, involved in visuospatial perception, ocular control, spatial attention and multimodal sensory integration [86]. FA reductions in the AMD group thus covered both distinct and common areas with those affected by atrophy (e.g., superior frontal cortex, and lingual and cuneate, occipital areas).

The occipital areas outlined in the second acquisition include the posterior occipital lobe in the primary visual cortex, and it is expected that changes in vision drive changes to the cortex [87]. This marked decrease in FA in comparison to a lesser decrease in volume in the occipital cortex gray matter may be specific to AMD as similar results have been observed for AMD when compared to juvenile macular degeneration [48]. This could also reflect neuroplasticity as loss of vision does not lead to permanent inactivation of the visual cortex, and there is a long-term reorganization with a potential increase in brain activity in the occipital visual cortex long after an impairment in vision [88,89].

The left frontoparietal cortex is important for verbal fluency, and changes to this area were associated with cognitive deficits seen in early Alzheimer’s disease, including spatial neglect [90,91,92].

Our findings of accelerated FA reduction in visual, temporal, and cingulate areas complement reports of altered functional connectivity [82,93] in the cingulate, and its altered role in memory related tasks in AMD [37]. Damage in both the anterior and posterior cingulate has been associated with apathy, akinesia, and increased stupor [94]. Meanwhile, the posterior cingulate is involved in cognition, especially attention, and visual attention [74], as well as internally directed cognition, and shows changes in ageing and neurodegenerative diseases like AD [74,95]. The posterior cingulate is a highly connected hub of the default mode network [96], and connectopathies affecting the posterior cingulate and hippocampus may provide sensitive biomarkers for incipient neurodegenerative diseases [97].

The temporal lobe saw an accelerated atrophy and FA reduction that was not symmetrical, with more profound changes in the left hemisphere. This is of interest as the inferior temporal cortex is responsible for processing visual information from the occipital cortex [98]. The superior temporal cortex is involved in visual spatial-based processing and object-centered spatial orientation [99]. Importantly, the left superior temporal lobe includes Wernicke’s area, involved in language and speech processing [100,101]. The lateralized findings in the brain do not correlate to lateralized visual deficits, as vision loss was symmetric in many participants and “worse eye” was almost evenly distributed between the left and right eye. Given that AMD has been associated with poor performance in language-related cognitive tasks [14], it stands to reason that the brain changes reported here, including a rapid deterioration in volume and FA in the temporal lobes, could play a role in the acceleration of cognitive decline. The superior frontal cortex is also involved in spatial cognition and working memory [102]. Our findings parallel a decline in expression of synaptic proteins in multiple brain areas in elderly subjects with early impairment in memory and language [103,104].

Our tractography and connectomics analyses underlined the importance of the connections between the lingual gyri of the two hemispheres, as well as between the lingual gyrus and the cerebellum. The length of the streamlines in these connections of interest in the AMD group was less than in the controls and decreased over two years in AMD. While the FA for the lingual connections between the two groups was not found to be significantly different when comparing the totality of the streamlines from each connection, specific high-density bundles were found to have higher FA in the control than AMD. The lower length of streamlines and FA decrease, the latter of which was also observed in the FA VBA, which could be related to the atrophy of lingual gyri alongside pericalcarine regions that can also occur due to retinitis pigmentosa [105].

The lingual gyrus has been linked to visual processing and memory [73], and decreases in functional connectivity in this region, alongside the anterior cingulate cortex, was also associated with Alzheimer’s disease [106]. Our results support a connection between macular degeneration and the integrity of the lingual gyrus, whose damage has been associated to declining memory [15]. Interestingly, in healthy subjects with high genetic risk for late-onset AD, there are significant correlations between retinal changes and brain areas closely related to AD such as the lingual and entorhinal cortex [107].

While the cerebellum has been historically associated with motor functions, it has more recently emerged as a modulator of language, learning, and memory processes, the right cerebellum in particular [108], though its exact dynamics and involvement remain a subject of debate [109]. The left cerebellum has also been linked to cognitive functioning [110]. Given AMD’s link with linguistic cognitive decline, the significant group differences in the length of the streamlines connecting the cerebellum to lingual gyri and the lower FA in specific bundles is particularly interesting to note. Our study supports a key role for the cerebellar connections, in particular lingual–cerebellar inter-hemispheric connections as they appear to be deteriorating in individuals with AMD, possibly in conjunction with visual–spatial learning and memory [111].

Several limitations may impact the interpretation of our results. First, the small sample size limited the power of our comparative analysis. Future studies with larger numbers of participants should consider stratifying subjects based on the laterality and stage of AMD. Secondly, our analyses do not incorporate behavioral variables and thus are unbiased; however, this means that the regions and networks identified cannot be directly linked to specific cognitive deficits, and our interpretations on their roles are relying on the published literature. Third, to facilitate the recruitment of this older and multimorbid population, the inclusion criteria were fairly broad; any future study should try and set specific criteria as to the type of AMD and the time since initial diagnosis to better compare the development of the condition from a specific timepoint. Since aged and diseased subjects may have lower FA, we have used the DWI images for registration. Finally, our image acquisition used anisotropic voxels to achieve sufficient SNR and keep scan time short; however, voxel-based analyses and tractography would benefit from using isotropic voxels. Accelerated imaging protocols using compressed sensing or multiband acquisitions may help improve future studies.

While at its simplest a connectome reflects the properties of pairwise connections with one single entry in the adjacency matrix, these values can be influenced by multiple phenomena, e.g., toxic insults, vascular trophic effects, changes in number of axons or their myelination, or other downstream effects of neurodegenerative processes. These can alter the numbers of streamlines and their properties, which are not necessarily constant along the connection length. We showed that bundle analyses improved sensitivity. Affine registrations allowed us to compare the shapes of the streamlines, while still permitting us to compare FA parameters along parametrized tracks. We recognize a diffeomorphic registration would increase the accuracy of the mapping but would limit the ability to compare shapes.

Future analyses should focus on the dynamics of connections between the superior frontal right and left regions, as it could be a particularly volatile region that sees important changes when AMD is already established. It might be also of interest to look into the evolution of the connections of the right inferior frontal gyrus and see whether changes in functional connectivity lead to structural reorganization and how the brain changes relate to cognitive decline. Future studies should also examine the asymmetry in neuronal changes when AMD affects the eyes nonuniformly.

Multiple theories can explain AMD-related brain changes beyond occipital areas, and these include secondary degeneration of possible visual areas or areas connected to them, brain plasticity and compensatory mechanisms in AMD, a co-existence with neurodegenerative processes, or a common mechanism and shared genetic risk. Our study shows that visual system changes are associated not only with changes in the brain regions involved in visual processing, but also with atrophy in areas involved in language and memory. Some of these changes were only observed at the 2-year mark, which supports the idea that the progress of AMD pathology is correlated with neurodegenerative conditions, which alters the brain aging trajectory.

## 5. Conclusions

Our results demonstrated that diffuse volume atrophy and microstructural changes in visual areas distinguish AMD participants from age-matched controls and that this effect grows greater over time. Moreover, some regions, which showed little difference from controls initially, showed a more rapid decline, including areas known to be involved in memory and language. Our longitudinal tensor network analysis revealed a clear pattern of AMD-related changes: not only does tractography seeded in visual cortex demonstrate a faster decline in white matter integrity in AMD, but there are more pronounced connectivity changes in regions linked to language, speech, and memory. Identifying specific patterns of regional atrophy and connectopathy may provide greater insight into the mechanisms associated with greater cognitive decline in AMD.

## Figures and Tables

**Table 1:** Top connections differentiating AMD participants from controls, at the initial visit and 2 years later. L: Left; R: Right.

	Connection	Average Number Streamlines Control	Average Number Streamlines AMD	T Values	*p* Values	FDR-Corrected *p*-Values
1st Visit	Insula R– Rostral Middle Frontal R	270	451	−5.85	1.24 × 10^−5^	0.041
	Rostral middle frontal R– Post Central R	47	76	−4.45	2.75 × 10^−4^	0.46
	Parsopercularis L– Lateral Orbito Frontal L	430	270	3.59	5.37 × 10^−4^	0.89
	Inferior Temporal L– Inferior Temporal L	1836	1377	3.57	2.04 × 10^−3^	0.89
2nd Visit	Lingual R– Lateral Occipital R	642	468	3.31	3.66 × 10^−3^	0.95
	Lateral Orbito Frontal R– Caudal Middle Frontal R	155	69	3.25	4.22 × 10^−3^	0.95
	Parsopercularis R– Lateral Orbito Frontal R	332	219	3.23	4.38 × 10^−3^	0.95
	Cuneus R–Superior Parietal L	57	123	−3.16	5.18 × 10^−3^	0.95

**Table 2:** Top 10 TN-PCA results differentiating AMD subjects from age-matched controls: (a) at the first visit; (b) at the second visit, two year later; (c) in the rate of change in connectivity.

	Index	Connections	Weight
(a) 1st Visit	1	Lingual Right–Lingual Left	1063.86
	2	Fusiform Right–Superior temporal Left	1019.53
	3	Superior frontal Right–Superior frontal Left	966.39
	4	Inferior temporal Right–Superior temporal Left	924.91
	5	Superior temporal Right–Superior temporal Left	888.21
	6	Fusiform Right–Insula Left	840.45
	7	Inferior temporal Right–Insula Left	823.66
	8	Insula Right–Superior temporal Right	801.33
	9	Superior temporal Right–Fusiform Right	776.92
	10	Middle temporal Right–Superior temporal Left	756.07
(b) 2nd Visit	1	Lingual Right–Cerebellum Cortex Right	1194.30
	2	Lingual Right–Cerebellum Cortex Left	1070.30
	3	Lingual Left–Cerebellum Cortex Left	986.70
	4	Lingual Right–Cerebellum Cortex Right	980.10
	5	Fusiform Left–Cerebellum Cortex Right	978.35
	6	Fusiform Right–Cerebellum Cortex Left	966.35
	7	Fusiform Right–Lingual Left	930.43
	8	Lingual Right–Fusiform Left	929.39
	9	Cerebellum Cortex Right–Cerebellum Cortex Left	925.83
	10	Lingual Right–Lingual Left	923.16
	9	Cerebellum Cortex Right–Cerebellum Cortex Left	925.83
	10	Lingual Right–Lingual Left	923.16
(c) Change Rate	1	Superior Frontal Right–Superior Frontal Left	4280.35
	2	Rostral Middle Frontal Right–Superior Frontal Left	3656.48
	3	Lateral Orbitofrontal Right–Medial Orbitofrontal Left	3218.92
	4	Medial Orbitofrontal Right–Lateral Orbitofrontal Left	2436.21
	5	Pre-Central Right–Superior Frontal Left	2070.61
	6	Caudal Middle Frontal Right–Superior Frontal Left	2057.30
	7	Superior Frontal Right–Paracentral Right	1965.98
	8	Medial Orbitofrontal Right–Lateral Orbitofrontal Right	1915.67
	9	Superior Frontal Right–Precentral Left	1842.59
	10	Lateral Orbitofrontal Right–Lateral Orbito-frontal Left	1674.94

**Table 3:** Tractography comparison based on length and FA along streamlines for three connections of interest.

	Connections	Group	Mean Length	Std Length	*p*-Val F-Val Cohen	Mean FA	Std FA	*p*-Val F-Val Cohen
1st Visit	Lingual Right–Lingual Left	Control	34	1.08	<0.001 108.2 0.45	0.109	0.004	0.0975 2.9 −0.10
		AMD	28	1.04		0.119	0.004	
	Lingual Left–Right Cerebellum Cortex	Control	66.6	1.73	<0.001 52.0 0.45	0.141	0.005	0.05 4.1 0.134
		AMD	54.7	1.74		0.127	0.005	
	Lingual Right–Left Cerebellum Cortex	Control	48.3	1.13	<0.001 15.7 0.17	0.115	0.004	0.40 0.7 −0.06
		AMD	45.4	1.1		0.119	0.004	
2nd Visit	Lingual Right– Lingual Left	Control	34.4	0.88	<0.001 290.5 0.68	0.105	0.006	0.27 1.2 −0.11
		AMD	25.9	0.82		0.114	0.006	
	Lingual Left– Right Cerebellum Cortex	Control	53.8	1.06	<0.001 130.8 0.35	0.130	0.005	0.37 0.8 0.07
		AMD	47.5	1.02		0.124	0.005	
	Lingual Right– Left Cerebellum Cortex	Control	59.2	1.31	<0.001 1330 1.08	0.128	0.005	0.12 2.6 0.13

**Table 4:** Similarity statistics for bundle sets were estimated based on distance, BUAN, coherence, length, and FA along tracts for bundles with highest FA difference and that has high spatial comparability. Avg: Average; Std: Standard Deviation; *p*-val, T-val: *p*-value, T value; bund: bundle; COI: Connections of Interest; BUAN: BUndle ANalytic values; Len: Length of streamlines; Coh: Coherence, Cereb: Cerebellum; R: Right; L: Left.

Year	COI	Group	Avg Dist	Std Dist	*p*-Val T-Val Cohen	Avg BUAN	Std BUAN	*p*-Val F-Val Cohen	Avg Coh	Std Coh	*p*-Val F-Val Cohen	Avg Len	Std Len	*p*-Val F-Val Cohen	Avg FA	Std FA	*p*-Val F-Val Cohen
1st Visit	Lingual R–Lingual L	CTRL	16.3	8.48	0.02 −2.36 −0.40	0.83	0.35	0.06 1.88 0.32	0.36	0.04	0.34 2.2 0.22	37	2.1	0.05 5.46 −0.34	0.22	0.01	0.28 2.49 −0.2
		AMD	20.0	10.04		0.71	0.41		0.28	0.04		43	2.1		0.24	0.01	
	Lingual L–Cereb Cortex R	CTRL	15.0	4.6	0.18 1.33 0.20	0.95	0.1	0.02 −2.33 −0.35	0.26	0.03	0.85 0.047 −0.03	83.7	4.33	0.0016 15.1 0.77	0.22	0.006	<0.001 40.4 0.63
		AMD	14.1	4.1		0.98	0.05		0.27	0.03		67.9	3.74		0.17	0.006	
	Lingual R–Cereb Cortex L	CTRL	16.8	6.8	0.84 −0.20 −0.03	0.86	0.29	0.52 −0.64 −0.096	0.30	0.03	0.89 0.089 0.034	58.5	2.3	<0.001 23.7 0.59	0.22	0.006	0.02 9.45 0.31
		AMD	17.0	6.60		0.88	0.22		0.29	0.03		45.7	2.2		0.19	0.006	
2nd Visit	Lingual R–Lingual L	CTRL	17.9	8.2	0.010 1.68 0.28	0.85	0.30	0.15 −1.43 −0.24	0.34	0.041	0.37 1.12 0.16	42.5	2.8	0.11 3.58 0.33	0.21	0.008	0.007 12.0 0.35
		AMD	15.7	7.4		0.91	0.21		0.28	0.044		36.2	2.9		0.17	0.009	
	Lingual L–Cereb Cortex R	CTRL	14.9	5.2	0.50 −0.67 −0.10	0.94	0.14	0.52 0.65 0.01	0.34	0.03	0.008 12.1 0.40	72.0	0.03	0.81 0.21 −0.07	0.19	0.005	0.01 11.2 0.39
		AMD	15.4	5.5		0.93	0.16		0.23	0.02		46.3	0.03		0.16	0.006	
	Lingual R–Cereb Cortex L	CTRL	16.4	5.37	0.45 −0.76 −0.11	0.92	0.16	0.066 1.85 0.28	0.25	0.03	0.98 0.26 0.09	92.1	3.4	0.002 14.6 0.69	0.23	0.005	<0.001 39.8 0.59
		AMD	17.1	6.73		0.86	0.27		0.23	0.03		71.5	4.4		0.18	0.006	

## Data Availability

Datasets with limited personal health information can be made available via a request to the authors, after establishing a formal data sharing agreement between Duke University and the recipient, a brief protocol, and approval from the requesting researcher’s local Institutional Review Board. Code link: https://github.com/JacquesStout/DTC_private/tree/main/AMD (accessed on 4 January 2024).
